# The Future of Biomarkers in Veterinary Medicine: Emerging Approaches and Associated Challenges

**DOI:** 10.3390/ani12172194

**Published:** 2022-08-26

**Authors:** Tharangani R.W Perera, David A. Skerrett-Byrne, Zamira Gibb, Brett Nixon, Aleona Swegen

**Affiliations:** Priority Research Centre for Reproductive Science, College of Engineering, Science and Environment, University of Newcastle, Callaghan, NSW 2308, Australia

**Keywords:** biomarkers, veterinary, proteomics, lipidomics, metabolomics, genomics, transcriptomics

## Abstract

**Simple Summary:**

In this review we seek to outline the role of new technologies in biomarker discovery, particularly within the veterinary field and with an emphasis on ‘omics’, as well as to examine why many biomarkers-despite much excitement-have not yet made it to clinical practice. Further we emphasise the critical need for close collaboration between clinicians, researchers and funding bodies and the need to set clear goals for biomarker requirements and realistic application in the clinical setting, ensuring that biomarker type, method of detection and clinical utility are compatible, and adequate funding, time and sample size are available for all phases of development.

**Abstract:**

New biomarkers promise to transform veterinary practice through rapid diagnosis of diseases, effective monitoring of animal health and improved welfare and production efficiency. However, the road from biomarker discovery to translation is not always straightforward. This review focuses on molecular biomarkers under development in the veterinary field, introduces the emerging technological approaches transforming this space and the role of ‘omics platforms in novel biomarker discovery. The vast majority of veterinary biomarkers are at preliminary stages of development and not yet ready to be deployed into clinical translation. Hence, we examine the major challenges encountered in the process of biomarker development from discovery, through validation and translation to clinical practice, including the hurdles specific to veterinary practice and to each of the ‘omics platforms–transcriptomics, proteomics, lipidomics and metabolomics. Finally, recommendations are made for the planning and execution of biomarker studies with a view to assisting the success of novel biomarkers in reaching their full potential.

## 1. Introduction

Veterinary medicine is a fast-growing field and arguably has effectively embraced cutting edge developments arising in other health fields, such as biomedicine. Veterinary professionals hold considerable responsibility for the quick diagnosis, treatment, disease prevention, and nutrition of animals, working towards both enhanced animal welfare and human public health outcomes [1,2]. Only a few decades ago, veterinary disease diagnosis mainly relied on clinical signs, with confirmation via a limited repertoire of laboratory tests and microbiological cultures, with imaging via radiography and ultrasound incorporated more recently. Such confirmatory diagnoses often take several days and may require outsourcing or referral and specialist expertise. This not only delays treatment but in the case of infectious diseases, time to diagnosis is critical as spread of a disease can result in mass culls and huge economic losses, with widespread implications for industry and public health [3]. To address those welfare and economic issues, routine, robust and early diagnostic tests are increasingly being developed and incorporated into practice. Thus, biotechnology is now playing a major role in the veterinary field, with potential applications extending to animal reproduction and the diagnosis and treatment of animal diseases [4]. While the utility of new biotechnologies for veterinary diagnostics is now recognised and there are several examples of successful incorporation into routine practice, there persist numerous challenges in biomarker identification and translation to clinical use.

The origins of the biotechnology field stem from the discovery of microbiology and cellular biology in the twentieth century, including the development of vaccines, sera and antibiotics, as well as selective breeding and cross-breeding of plants and animals. At this time, the detection of pathogens also became possible, mainly based on microbial culture methods, biochemical tests, and microscopy. These methods can be time consuming and require specific equipment and expertise, driving a need for rapid, accurate and sensitive diagnostic tests to detect disease pathogens [1,4]. More recently, with the development of molecular biology, the realm of biotechnology has expanded greatly to include DNA manipulation, gene engineering [5] and a host of research, agricultural and medical applications. A major milestone was the sequencing of the human genome. Along with ongoing characterisation of genomes of other species, this has facilitated an era of biotechnology where mass spectrometry (MS)-driven “omics” platforms—proteomics, lipidomics, metabolomics, transcriptomics, epigenomics and genomics—allow the generation of extensive datasets in almost any tissue, cell or species [6]. These new technologies are continuously improving in capability and becoming more accessible, revealing vast amounts of information about the molecular properties of biological systems [4,6].

The use of biomarkers has been identified as an increasing trend in the animal health industry and has been applied to the evaluation of a variety of health parameters. In particular, it is useful in clinical applications such as diagnosing illness, predicting and/or tracking the response to treatment, and determining the toxicity or failure of an organ. The term biological marker first appeared in the literature in the late 1960s [7]; the term “biomarker” is a shortened version that became more commonly used by the 1990s, albeit in various and often inconsistent ways [8]. Though there are several definitions for the term “biomarker” in human medicine [7,9,10,11], the most relevant and applicable to veterinary medicine is the BEST glossary broadened definition of biomarkers and related terms by the animal health industry [8]. The definition is “A defining characteristic that is measured as an indicator of normal biological processes, pathogenic processes, or biological responses to an exposure or intervention, including therapeutic interventions”. This definition classifies biomarkers into types as “molecular, histologic, radiographic, or physiologic characteristics”. Further, seven categories of biomarkers are proposed: susceptibility/risk biomarker, diagnostic biomarker, monitoring biomarker, prognostic biomarker, predictive biomarker, pharmacodynamic/response biomarker, and safety biomarker [12]. In this review, we focus on molecular biomarkers, and in particular, the role of emerging technological platforms in biomarker discovery and translation.

Opportunities to develop new biomarkers can arise in different ways; for example, a planned search for a biomarker for a specific disease, a chance finding of a marker (protein, lipid, metabolite, etc.) that could be used as the target for a new biomarker test, or a comparison to existing biomarkers in human medicine [13,14]. Many of the biomarkers typically included in standard haematology and biochemistry profiles have been known for many years and were adapted from human medicine [15,16,17]; the same applies for many of the more recent additions to molecular diagnostics, as discussed later in this review. With the advent of new technologies, it is now possible to conduct large-scale studies directed specifically at identifying novel biomarkers capable of predicting or diagnosing a given condition. Currently, biomarker discovery in the veterinary field is increasing, in tune with a new era of precision and high-quality practice in veterinary care. While older biomarkers tend to focus mostly on diagnosing disease processes and organ dysfunction, more recently there is an increasing trend in identifying markers for survival and response to treatment [8]. This is critical in cases where a disease or outcome is relatively uncommon but poses a significant medical challenge because there is currently no method for early detection of those diseases, and they are typically not diagnosed until patients have progressed to the symptomatic stage, which reduces the chances of survival and reduces treatment efficacy [18]. Beyond pathological conditions, this also applies to other biological processes of importance in animal management, reproduction, and husbandry, all of which contribute to good animal welfare and public health. Therefore, we consider it is important to examine the process of biomarker invention.

**Figure 1:**
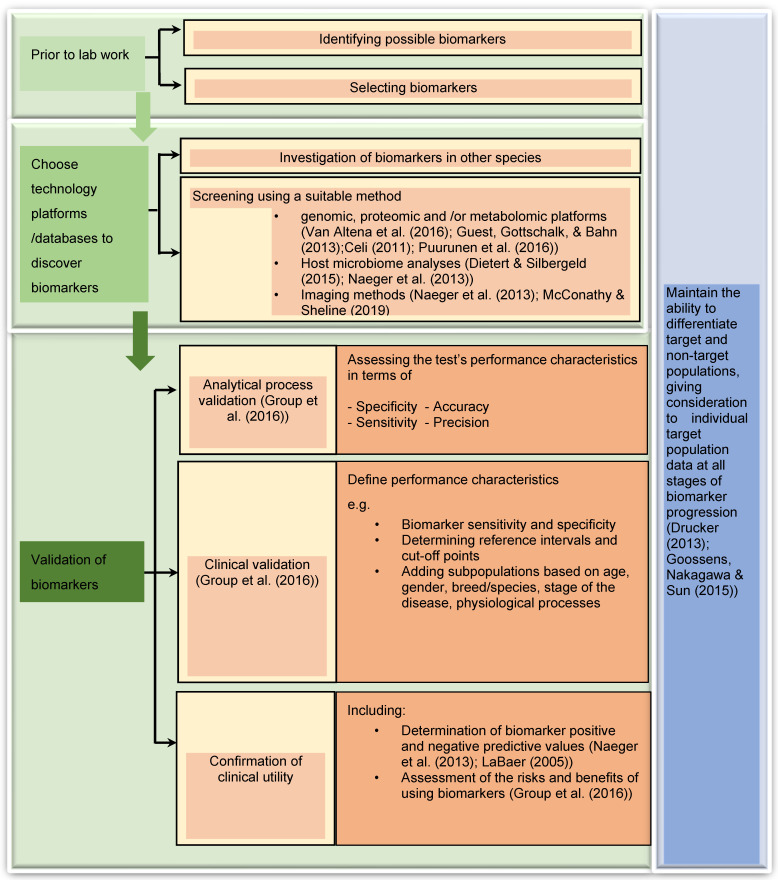
Visual summary of proposed workflow for effective biomarker discovery and validation as discussed in this review [10,23,24,25,26,27,28,29,30,31,32,33].

According to the review of Myers et al. [8], the most successful biomarker research process involves first clearly identifying the clinical utility and target population, then working backward and again forward in sequential steps to select and validate the putative biomarker(s) along with the related analytical procedure(s). The flowchart given in Figure 1, illustrates the steps involved in this process. Specifically in the veterinary field, new diagnostic biomarkers are being developed for the early detection of disease, when it may be reversible or more easily managed, and susceptibility/risk biomarkers are being developed to identify animals with increased resistance or susceptibility to disease [8]. To develop these tests, blood, and body fluids such as urine, saliva, endometrial fluids are utilised most often. As technological capabilities expand, molecular markers within individual cells are also becoming feasible. When challenged with a particular biological change or disease, somatic cells secrete specific substances to the extracellular tissue or body fluids. As such, changes in the concentration of the substance of interest can be measured from those samples. Blood plasma tends to contain transudates from almost all kinds of tissue as it circulates the entire organism and can be easily collected from the patient. For these reasons, easily and routinely obtained samples from the patients such as blood plasma and serum are most commonly used for diagnostic purposes [19,20,21].

A range of techniques are currently being used to identify novel biomarkers and validate their predictive value, ranging from the more established science of enzymatic tests to the emerging technology of nanoscience and “omics” approaches that encompass multivariate large-scale analysis at the level of DNA, miRNA, RNA, proteins, lipids, and other metabolites. These techniques take a snapshot of the current state of cells, tissues, or bodily fluids and find the best representative marker for the condition [22]. The omics platforms are a relatively recent addition to the biomarker discovery toolset, and whilst they have facilitated remarkable discovery power in identifying potential biomarkers, the majority of these have yet to become routinely used, widely available clinical tools. In this review, we examine the roles of the omics platforms in veterinary biomarker identification, and the challenges that lay on the path from discovery to clinical application. 

## 2. The Omics Platforms and Their Role in Biomarker Discovery

### 2.1. Genomics

Although many factors influence one’s health and sickness, it is apparent that genetic heritage is a significant contributor. Examining this genetic background is crucial for discovering particular mutations and/or variations that underpin pathways that differentiate between health and sickness [6,34]. Further, both illness risk and treatment response are influenced by genetic variation. Genome-wide association studies have enabled the identification of genetic variants that contribute to the pathophysiology of complex genetic diseases [35], as well as the detection of several pharmacogenetic markers [36,37]. A genome-wide association study (GWA study, or GWAS), also known as whole genome association study (WGA study, or WGAS), is an observational study of a genome-wide set of genetic variants in different individuals to detect if any variant is associated with a trait of interest.

DNA biomarker tests, for example, can be used to decide if treatment can be safely postponed for a time of watchful waiting in the case of prostate cancer. If the tumour is found to be devoid of genes that cause an aggressive form of cancer, it may remain stable for decades, obviating the need for major surgical removal followed by radiotherapy or chemotherapy. Genetic profiles, on the other hand, may also be utilised to determine preventive treatments [38]. Individual genetic testing is used to decide on specific, sometimes extremely radical interventions such as prophylactic surgery in some cases of hereditary cancer [38]. Biomarker based on DNA Single Nucleotide Polymorphism (SNPs), Short Tandem Repeats (STRs), deletions, insertions, and other DNA sequence variations are examples of germline biomarkers. Compared to expression-based indicators, DNA methylation biomarkers have numerous important advantages. For example, even if changes are present in a small number of cells, they are easily amplifiable and detectable using polymerase chain reaction (PCR)-based techniques [39]. DNA methylation is a highly stable marker that may be identified in a wide range of minimally invasive materials, including saliva, plasma, serum, urine, sperm, and faeces [40]. Many DNA biomarkers for cancer in human medicine are now commonly used in commercial test kits, highlighting their successful application after initial identification as biomarkers [41,42]; e.g., VIM gene (vimentin) is used for diagnosis of colorectal cancer in a commercially available kit known as “Cologuard” and marketed by Exact Sciences [43].

According to a review of the literature by Myers, Smith and Turfle [8], the most recent significant advances in disease susceptibility/risk, diagnostic, and prognostic biomarkers for veterinary clinical medicine have occurred in the areas of genomic markers of disease resistance or susceptibility, as well as diagnostic, prognostic, and monitoring biomarkers for kidney disease, cardiovascular disease, stem cell biology [44], and cancer. The genome sequencing of domestic animals, such as the horse, cow, dog, and cat has aided rapid progress in the identification of the genetic basis for disease susceptibility and resistance [8]. Disease genetic markers are fast being identified, and genetic testing for determining disease risk in both companion and livestock species is becoming available. The discovery of genetic markers linked to the development of mast cell malignancies in golden retrievers [45], dilated cardiomyopathy in Doberman pinschers [46], lavender foal syndrome in Arabian foals [47], and cholesterol insufficiency in Holstein cattle serve as a few recent examples [48].

The availability of tests to discover genetic biomarkers of disease has the potential to improve the health of companion and livestock animals and influence breeding decisions, as well as contribute to a better knowledge of diseases that affect humans [49]. For livestock species, genetic indicators of immune system function and infectious disease susceptibility are being created, which should improve animal health and potentially reduce dependency on some therapeutic medications, such as antimicrobials [50]. In the United States, progress has been made in identifying genetic biomarkers of vulnerability to bovine respiratory illness, one of the most frequent (and possibly fatal) infectious diseases in cattle [51].

DNA sequencing technologies are well-established meaning that genetic biomarkers benefit from a straight-forward validation process and relatively low costs of assay development once a suitable biomarker has been identified. Requirements for sample drawing, handling, and storage are minimal, and equipment for testing tends to be widely available. On the other hand, genetic biomarkers are mostly limited to screening tests and general predictions about disease susceptibility rather than more specific clinical endpoints and cannot be used in more dynamic situations since they are limited to examining the animal’s static genetic code. Thus, biomarkers emanating from the newer ‘omics platforms have greater scope for generating an accurate and specific profile of health or disease. Nevertheless, continued evolution in the sensitivity and depth of sequencing technologies and ongoing investment into GWAS will undoubtedly continue to generate efficient genetic tests to improve animal welfare, particularly at the population level. 

### 2.2. Transcriptomics

The transcriptome lies immediately downstream of the genetic code and captures a more responsive state than does DNA sequencing, by providing a profile of genes being actively transcribed and regulated at a given timepoint. The transcriptome consists of all the total complements of ribonucleic acid (RNA) transcripts in a cell, tissue or body fluid [6], comprising both coding and non-coding RNAs. Of total RNAs, coding transcripts (messenger RNA; mRNA) comprise 1–4% while non-coding transcripts make up the remaining >95% and include ribosomal RNA, transfer RNA, small nuclear RNA, small interfering RNA, microRNA and long-non-coding RNA [14,33,34]. RNA reflects cellular states by delivering genetic information and regulatory information by transcriptional and post-transcriptional regulation [52,53]. Rapid advances in RNA biomarker research have led to creation of a significant variety of high-performance RNA-detection technologies in recent years [54]. Molecular biology techniques such as quantitative reverse transcription polymerase chain reaction (RT-qPCR) [55,56], microarrays [57], and RNA sequencing [58] are the methods used in these technologies. Next-generation sequencing technology has recently made it possible to quantify RNA expression levels at the full genome level. Increasing the depth of RNA sequencing now facilitates the detection of novel transcripts, such as lowly expressed noncoding RNAs, and their modest expression fluctuations, with high accuracy [59,60]. RNA biosensors, micro- and nanofabrication technologies, and diverse readout techniques, such as electrochemical and optical transducers, have all received a lot of interest in recent years [54].

The first well-studied type of RNA as biomarker is mRNA [61]. The study of mRNAs provides direct insight into the gene expression characteristics of individual cells and tissues. It allows the measurement of the presence/absence and quantification of a transcript, assessment or prediction of protein isoforms and quantitative assessment of genotype influence on gene expression using expression quantitative trait loci analyses (eQTL) or allele-specific expression [6]. Accumulating data based on high-throughput sequencing technology shows that diverse RNA molecules can serve as biomarkers for the diagnosis and prognosis of various diseases, such as cancer [54]. Many cancer studies have explored multi-gene expression patterns as a biomarker for clinical outcome [62]. PAM50, for example, is a 50-gene panel that has been successfully used to classify breast cancer [63]. Another expression panel of 31 mRNAs linked to cell cycle progression was employed as a prognostic marker to predict prostate cancer metastasis, recurrence, and risk [64]. 

In addition to mRNA, certain miRNAs play critical roles in cell proliferation, differentiation, and death, and hence act as tumour suppressors or oncogenes [65]. It has been observed that miRNA expression profiles can successfully distinguish poorly differentiated tumour types [66]. Furthermore, reduced *miR-21* expression was linked to a lower hazard risk in individuals with pancreatic ductal adenocarcinoma after adjuvant therapy. Moreover, *miR-21* has been identified as a possible therapeutic target. Extracellular RNAs (exRNAs) are emerging as non-invasive biomarkers for earlier cancer diagnosis, tumour progression monitoring, and therapeutic response prediction, as they are detectable in diverse bio-fluids such as serum, saliva, and urine [67]. 

RNA biomarkers are also valuable in distinguishing various diseases in veterinary medicine. Dirksen, et al. [68] showed the ability to distinguish between parenchymal, biliary, and neoplastic hepatobiliary diseases using a panel of microRNA consisting of *miR-21*, *miR-122*, *miR-126*, *miR-200c*, and *miR-222*. Lecchi, et al. [69] have identified miRNAs can serve as potential biomarkers for *Brucella* infection in water buffaloes (*Bubalus bubalis*). The identified miRNAs were involved in regulating the transcription of genes related to the molecular pathogenesis of brucellosis. Further, they have identified *miR-let-7f*, *miR-151*, *miR-30e*, *miR-191*, *miR-150* and *miR-339b* extracted from vaginal fluids, which are potentially useful biomarkers of *Brucella* infection. miRNAs also appear to be a very useful tool in identifying different disease conditions such as osteochondrosis, rhabdomyolysis, insulin resistance and osteoarthritis [70,71,72] according to the functional role of the identified miRNAs in corresponding healthy tissues in horses [73,74]. 

In contrast to genome sequencing technology, accurate determination of RNA levels often requires some form of amplification, a time-consuming sample pre-treatment procedure, and the associated expense of appropriate equipment. Working with RNA is more demanding due to its chemical instability, with the geometry of the molecule as a single stranded polynucleotide being very susceptible to degradation reactions. Therefore, RNA can easily be affected by oxidation and spontaneous changes of phosphodiester linkage through transesterification. Special precautions must be taken when working with RNA to address the high risk of contamination such as the ubiquitous presence of RNases [75]. Furthermore, because oligo(dT) primers are used for amplification, they frequently cannot amplify RNA sequences without a poly(A) tail [76]. Moreover, to prevent sample heterogeneity caused by physiological and systemic changes in clinical samples, very high sample volumes are required for sensing RNA biomarkers in body fluids [77]. The storage of the samples is generally done at −20 °C, −80 °C or under liquid nitrogen, thus requiring more sophisticated facilities for storage, transport and processing of samples [75]. Several nanotechnology-based RNA sensing systems linked with optical and electrochemical readouts have been developed as a result of extensive study to uncover relatively robust, accurate, and effective methodologies. These methods provide simple sampling processes, quick and cost-effective analysis, portability, label-free and amplification-free choices, and portability [78,79,80]. Electrochemical approaches, for example, have demonstrated ultra-high sensitivity and selectivity, as well as a high potential for multiplexed analysis in a point-of-care platform [81,82]. However, the functioning of electrochemical RNA sensors is still limited to proof-of-concept research, and various obstacles must be overcome before these technologies may be used in typical clinical settings [54,76].

Although serum circulating RNAs are considered some of the most promising clinical diagnostic or therapeutic biomarkers in both humans and animals, their diagnostic potential in veterinary medicine remains to be fully explored [69]. Many of these studies provided new and important insights into disease pathogenesis and further experiments involving more animals are required to validate the potential use of RNAs in clinical diagnostics.

### 2.3. Proteomics

Proteins are the ultimate endpoint of transcription and translation of the genetic code and are the workhorses of cells and tissues as they are responsible for carrying out the biochemical and metabolic functions required for survival and homeostasis. This includes the response to disease and various physiological states. A remarkably useful feature of this biological cascade is that each protein is directly encoded by a corresponding gene and can thus be unambiguously traced to a unique identifier based on its amino acid sequence. Proteomic technologies, i.e., mass spectrometry (MS) and its adjuncts, take advantage of this feature to generate profiles of hundreds to thousands of proteins reflective of specific cells, tissues, fluids or biological states, known as proteomes. Many animal researchers [83,84,85,86] are now exploiting the advances in proteomics [87,88] and directing its use to biomarker discovery in the future. Being the phenotypic endpoint of any given biological process, proteomes not only reveal mechanistic information about how biological responses operate but also reveal high resolution, detectable differences that can be used as specific biomarkers or treatment targets [89].

In proteomics, quadrupole mass spectrometry is most commonly used in conjunction with time-of-flight (TOF) or Orbitrap analysers. Proteome identification and quantification, protein–protein interactions (interactomics), organellar proteomics, post-translational modification detection, and many more applications have now become possible with advances in MS. Accordingly, this field is now well positioned to contribute significantly to translational medicine, notably in the identification and routine use of biomarkers. Although MS-based proteomics is more sophisticated than antibody-based techniques, it exhibits exceptional specificity of detection, and allows large scale screening and hypothesis-free exploration to identify novel biomarkers. Meanwhile, antibody techniques remain most accessible when seeking to detect a specific protein already identified as a biomarker or protein of interest. The sensitivity of MS processes has improved dramatically, allowing single-cell proteomics to become a reality. Proteomics has the extra benefit of allowing researchers to study single cells while keeping the full spatial information of the cellular environment. Furthermore, when compared to their equivalent mRNAs, there are far more protein copies, making single-cell proteomics intrinsically more robust. Intercellular dynamics such as receptor–ligand interactions between cells and their surroundings will be immediately revealed by MS-based single-cell proteomics [89]. These techniques of single cell proteomics will lead to identify pathways that are activated in therapy-resistant cells and can provide biomarkers for cancer diagnosis and for determining patient prognosis.

Liquid chromatography and MS- based proteomics approaches are driving the development of novel veterinary biomarkers [90]. Biomarkers linked with canine babesiosis, such as apolipoproteins and vitamin D metabolism-related proteins, have been detected in dogs [91]. These same biomarkers alongside several new possible proteins for treatment monitoring of canine leishmaniosis have been reported in another investigation [92]. These biomarkers would be advantageous in a clinical setting because they are expected to be quicker than the conventional diagnostic methods such as identifying organisms in blood smears; it may also be possible that testing could be conducted in general practice without the need for an expert to identify the organism in the blood smear. Treatment monitoring biomarkers will help the owners to manage animals at home and inform the vet rather than having the animal stay in hospital for monitoring, reducing stress and costs of aftercare. Proteomic studies on feline biomarkers have also been conducted in research on congestive heart failure due to primary cardiomyopathy [93]. The application of proteomics in farm animal health has sparked considerable interest in biomarker research, notably for subclinical but economically important diseases such as bovine mastitis, where an on-farm biomarker test could be quite useful [94]. Tandem Mass Tag (TMT) technology was applied for the first time for protein quantification using saliva to uncover new biomarkers for stress in sheep, revealing six proteins as potential markers, including those associated with hyperglycaemia—an immediate physiological response to stress [95]. In another application of proteomics, the cause of mortality in manatees following two separate mortality episodes was explored using a combination of 2D-DIGE (two-dimensional difference gel electrophoresis) and shot gun proteomics in which isobaric tags for relative and absolute quantification (iTRAQ) LC–MS/MS were used; with both techniques yielding similar results including an increased quantity of complement C4 protein, which was subsequently validated by immunoblotting [96]. To find biomarkers for early and late-stage oral melanoma, benign oral tumours, oral squamous cell carcinoma and periodontitis in dog using saliva, Ploypetch, et al. [97] used MALDI-TOF MS and LC-MS/MS and validated the markers using immunoblot analysis. One of the identified proteins was sentrin-specific protease 7 (SENP7), the expression of which significantly increased in oral squamous cell carcinoma. Expression of TLR4, was also increased in late oral melanoma and oral squamous cell carcinoma, compared with the control group. These studies represent but a few examples of active proteomic research directed toward development of biomarkers with significant welfare and economic impacts in the veterinary field. While the identified protein markers show potential for rapid diagnosis of the respective conditions, they are yet to be developed into clinically useful and commercially available diagnostic assays. 

While proteomic platforms such as MS provide unparalleled capacity for discovery and screening, validation still largely relies on antigen detection (immunoassay) techniques. The latter also tend to be most practical in clinical scenarios. As such, numerous immunoassay driven biomarkers have been developed or adapted from human medicine, including for renal disease, cardiovascular disease, and cancer. In veterinary cardiology, for example, for the detection of primary heart disease or myocardial damage secondary to other diseases, N-terminal pro-b-type (or brain) natriuretic peptides, and cardiac troponins have shown diagnostic and prognostic clinical utility across a broad range of animal species [98,99,100,101,102]. Acute phase proteins (APPs) are also becoming more popular as indicators in canine medicine, with Soler, et al. [103] demonstrating this principle with the development and validation of two enzyme linked immunosorbent assay (ELISA) techniques for assessing ITIH4 and haptoglobin (Hp) in dogs. 

The most simple and common antigen detection technique is the ELISA [104,105]. One prominent example is the recently developed bovine Pregnancy Associated Glycoproteins (PAG) ELISA to detect pregnancy in cattle. Since the ELISA is more efficient than previously existing Radioimmunoassay (RIA), the practicability of the PAG test was greatly enhanced. Friedrich and Holtz [106] have discovered a competitive double antibody ELISA using a polyclonal anti-PAG-IgG and an anti-rabbit-IgG raised in sheep for coating and application of newly established ELISA to test its suitability for pregnancy detection by measuring PAG in serum or milk. In their study, the ELISA proved to be an adequate and efficient way of measuring PAG in maternal serum or milk and was a useful means of pregnancy detection in cows.

Proteomics is currently at the forefront of biomarker development in both medical and veterinary fields, and benefits from many well-established protein detection methods that can be used downstream of discovery in clinical settings. While many proteins already serve as useful clinical biomarkers, the path from discovery to application is not always straightforward and some of the relevant challenges are discussed in subsequent sections of this review. 

### 2.4. Metabolomics

The term metabolomics became popular at the end of the 1990s to describe approaches that aim to measure all the low molecular weight metabolites present within a cell, tissue or organism during a genetic modification or physiological stimulus [107,108]. Proton nuclear magnetic resonance (1H NMR) spectroscopy, gas chromatography–mass spectrometry (GC–MS), and liquid chromatography–mass spectrometry (LC–MS) have all been employed in conjunction with pattern recognition algorithms in this process [109]. In less than two decades, proteomics and metabolomics have emerged as the functional continuation of transcriptomics and have progressed swiftly thanks to advances in technology and bioinformatics tools. While the proteome describes the set of enzymes, receptors and other machinery responsible for carrying out biochemical processes, the metabolome depicts the endpoint of those biochemical processes. Importantly, the metabolome may be a more accurate molecular depiction of phenotype and current state of health or function than any genome, transcriptome, or proteome-based biomarker because of its close relationship with phenotype and its real time reflection of the state of health of a patient [110]. Metabolites change faster and more dramatically than do genes or proteins, and those changes may be quantified in absolute terms, but genes and proteins show activity changes in a different way than concentration changes. Further, as metabolites can be assigned to metabolic pathways, their changes can usually be explained physiologically, enhancing their value.

In dairy cattles, biomarkers are being sought for dysfunction in key metabolic phases, for example the ‘transition period’ [111]. This is the period three weeks prior to calving and the three weeks following calving and is associated with major metabolic changes that transition from pregnancy to lactation. A compromised transition period can have significant detrimental impact on both welfare and productivity, while the molecular basis of effective versus compromised adaptation to the metabolic strain of early lactation remains unknown. Several dairy cow investigations have used targeted metabolomics to evaluate the respective changes in blood throughout the transition phase [112,113] in order to understand the pathophysiology and to identify promising biomarkers. Carnitines have been identified as possible biomarkers for metabolic illnesses associated with the transition period [112,114]. Hailemariam, Mandal, Saleem, Dunn, Wishart and Ametaj [112] suggest that the biomarker profiles they found (carnitine (C0), propionyl carnitine (C3), and lysophosphatidylcholine acyl C14:0 (lysoPC a C14:0)), such as any other set of candidate biomarkers, need to be further verified using a much larger cohort of animals to confirm their reliability. 

Using non-targeted metabolomics Wu, et al. [115], found leukotriene C4 (LTC4), leukotriene D4 (LTD4), chenodeoxycholate, linoleate, and other metabolites to be significantly different in a *Mycoplasma gallisepticum* (MG) and *Escherichia coli* (E.coli) co-infection model in serum. LTC4 in serum has been found as a potential biomarker for identifying poultry respiratory illness. Furthermore, to identify the consequences of co-infection, an arachidonic acid (AA) metabolic network pathway with metabolic products and enzyme genes were created and showed a similar dramatic increase in LTC4 expression, which was linked to varied degrees of infection. 

As discussed above, metabolomics is expected to enhance the accuracy of diagnosing the health status of patients by offering up reliable biomarkers. The expansion of the dynamic range of detection of low-abundance metabolites, in conjunction with the advent of artificial intelligence, will eventually pave the way for the detection of metabolic signatures as biomarkers; however, in the near future it is expected that such diagnostics will remain the domain of centralised facilities rather than point-of-care testing. 

### 2.5. Lipidomics

Lipidomics is a discipline concerned with the study of lipids, not only in terms of their structures and transformations, but also in terms of their diversity and activities in relation to cellular, metabolic, and environmental factors that influence living organisms [116]. Lipids represent major components of the cell membrane and are involved in a range of biological processes (organ function, metabolism, inflammation, endocrine signalling, etc.). Therefore, ratios of certain lipid species or classes are likely to be altered in response to pathological conditions and various physiological states, potentially serving as sensitive biomarkers. Indeed, lipids already form a useful component of clinical veterinary pathology, with cholesterol and triglycerides a mainstay of routine biochemistry analyses and extensively characterised for their association with a range of pathological and physiological processes. These lipids are readily measured in serum, plasma or cells using enzymatic reaction kits coupled with spectrophotometric detection. Newer analytical approaches are now allowing much higher resolution detection of lipid species, down to the lipid ion level, thus opening up potential for lipidomic signatures to serve as specific biomarkers.

Traditionally, three defined analytical approaches have been identified in lipidomics: direct infusion shotgun approaches, chromatography-based separation approaches, and imaging mass spectrometry. Within these approaches, the terms “untargeted” and “targeted” also describe how the decided analytical output influences the sample preparation, methodological technique, and data processing. There are also newer categories such as ‘macrolipidome’ and ‘microlipidome’ that refer to the particular lipid classes’ abundance and activity [117].

While the proteomes and transcriptomes of many pathologies and physiological states, tissues, and organs have already been mapped, the field of lipidomics is younger and fundamental descriptive studies to characterise normal lipidomes are currently underway. These will be crucial for providing the foundations and data repositories needed before biomarkers can be sought to differentiate disease states from normal physiology. Nonetheless, altered lipid levels have been identified in numerous diseases in both model animals and humans, e.g., differential fatty acid levels were identified in kidney [118] and liver [119] diseases, with such changes associated with other biochemical indicators. Lipid biomarkers are being sought for a range of veterinary applications, including predictors of wildlife mortality events [120], livestock production efficiency markers [121], early detection of subclinical disease and monitoring of drug toxicities [122]. 

MRM-profiling has been used to detect changes in the lipid composition of the epidermis in atopic dogs even when the skin seems clinically healthy, and sex is a modifying factor in the lipid profile of canine atopic dermatitis [123]. This study contributes to a better understanding of epidermal lipid alterations with the development of atopic dermatitis and as the chronic inflammatory process progresses. The high prediction rate for disease development provided by the lipid biomarkers found here by the machine learning technique suggests that they could be used as a molecular evaluation tool for atopic dermatitis diagnosis and monitoring, as well as patient response to treatment.

In milk obtained from cows with subclinical mastitis, untargeted lipidomics analyses revealed 597 lipids to be altered in abundance more than 10-fold versus non-infected samples [124]. Principal component analysis demonstrated distinct clustering based on both lipid class and lipid species between infected and healthy samples, although data are considered preliminary due to small sample size. It is also yet to be determined whether lipidome changes precede infection or are a consequence; nonetheless once identified, such markers could serve to indicate at-risk animals or those requiring further investigation.

Additionally, in cattle, several studies have revealed the potential of specific lipid classes for the identification of peripartal metabolic dysfunction [125,126]. Certain phosphatidylcholines appear to distinguish between healthy cows or those manifesting clinical metabolic disease during the periparturient period [125]. LC-TOF MS lipidomic profiling of plasma from cows affected by hepatic lipidosis (fatty liver disease), which also commonly affects dairy cows in transition from pregnancy to lactation, revealed a distinct plasma lipidomic profile with reduced phosphatidylcholines [127]. Diagnosis of fatty liver disease currently requires confirmation through biopsies to determine the hepatic lipid content, so a plasma biomarker could be extremely useful. Further investigation is needed to identify and validate specific phosphatidylcholines that could serve as a practical diagnostic tool for this disease. 

Lipidomic profiling has been applied in the investigation of wildlife mass mortality events; remarkably, adipose tissue collected from Mozambique tilapia (*Oreochromis mossambicus*) affected by pansteatisis (an environmentally derived inflammatory disease) showed up to a 1000-fold increase in ceramides and correlated with disease severity [120]. This highlights the potential utility of lipidomic biomarkers within biopsy or post-mortem-collected tissue samples in addition to fluids such as plasma.

Untargeted lipidomics has also been successful in profiling the lipids in plasma and urine of cats treated repeatedly with meloxicam, a non-steroidal anti-inflammatory (NSAID), thus identifying putative biomarkers for monitoring the effect of NSAIDs [121]. Here, 6 lipids in plasma and 5 in urine could discriminate meloxicam-treated from saline treated-cats. This work may ultimately lead to feline-specific pre-clinical biomarkers of NSAID-induced toxicity and would assist clinicians make therapeutic decisions according to individual needs, including selecting optimal dose intervals, thus minimizing the risk of adverse effects.

Another preliminary study identified lipidomic signatures that could potentially be used as a proxy for digestive efficiency in chickens, with important potential consequences for livestock production efficiency and economic output in the poultry industry [122]. However, as with many lipidomics studies, the exact chemical nature of the markers remains unconfirmed, and they are yet to be validated in an independent population. Notably, the authors report that the lipidomic investigation was triggered by an observation of differences in serum colouration between the two lines of broilers representing different digestion efficiencies. Indeed, a spectrophotometric analysis showed difference in absorption between 430 nm and 516 nm, corresponding to the signature of orange–red lipophilic pigments. Such observations tentatively promise innovative yet simple downstream applications of lipidomic biomarkers where incidental properties of certain lipid classes may facilitate means of detection that do not rely on the complex analytical methods used to identify the biomarkers in the first place. 

In the horse, elevated cyclic phosphatidic acid and diacylglycerol have been detected in surfactant from severely asthmatic horses using shot-gun lipidomics and suggested as useful biomarkers of this important inflammatory condition [128]. Yet again, these data can only be considered preliminary, and it is currently unknown whether the plasma lipidome is similarly altered in affected horses. 

Lipid biomarkers have long been a routine component of clinical diagnostics in veterinary medicine, and discovery of new markers via innovative lipidomics platforms is on the horizon. There is clear evidence for the association of specific lipid profiles with disease and physiological states; at present these are yet to be translated into practical and widely used diagnostic biomarkers. The putative lipid biomarkers described here are a long way away from widespread application, limited by the preliminary nature of most datasets, lack of standardisation in the metabolites and diseases studied, and few studies characterising the normal lipid profile in different physiological states and across species. Maturation of the lipidomic platforms, particularly with regard to standardised methods of analysis and interpretation of data, as well as expansion of publicly accessible data repositories, will facilitate efficient translation, as discussed further in this review.

### 2.6. Multiomics

A rapidly developing new approach for biomarker discovery is that of using multi modal integration. Omics technologies, as previously described, measure all or nearly all incidences of the targeted molecular environment in the assay, offering comprehensive perspectives of the biological system because they are high-throughput biochemical assays that evaluate molecules of the same type from a biological sample comprehensively and simultaneously. Initially, omics research focused on a single type of assay and produced single-omics results. However, more recently, researchers have designed multi-omics datasets by combining different assays from the same set of samples [129]. Because multi-omics data obtained for the same set of samples can reveal valuable information about the flow of biological information across many levels, it can aid in the understanding of the mechanisms behind the biological state of concern [130]. The advent of numerous new multi-omics projects has been fuelled by the constrained findings of early single-omics studies, such as the Human Genome Project, and the expansion of facilities that offer omics tests as a service. Identifiers from several timepoints in one or more omic types, phenotypic details such as treatment/control labelling, and pertinent clinical characteristics such as age and sex may all be included in multi-omics data. These findings give a comprehensive picture of disease-driven biological pathway dysregulation, as well as early proof for the establishment of new targets or intervention techniques [131].

Multi-omics’ increased potential has been evident for some time, but the challenge of maintaining and integrating such multi-dimensional data remains problematic. For big datasets, data storage, quality control, and statistical analysis are all more difficult, therefore adhering to the FAIR principles is naturally incredibly hard. Furthermore, creating full multi-omics datasets with the same set of omics tests for all research samples is an extensive undertaking. As a result, researchers who want to make use of these datasets’ multiplatform nature frequently have difficulty getting entire data records or finding appropriate multi-omics datasets for their research topics. Additional quality control metrics that analyse the link across datasets should be explored in the case of multi-omics data. These extra quality indicators are important since omics technologies differ in terms of accuracy, technical noise, and signal dynamic range, thus reliable integrative analytic results can only be drawn when quality is similar across platforms [129].

Omics data can be integrated in a variety of ways after pre-processing. Broadly there are two main approaches. One is post integration where each ‘omic’ approach can be analysed or modelled independently and then the findings integrated; the other is prior integration where data for all omic modalities can be integrated before any statistical or computational modelling takes place. Data may need to be prepared differently depending on which integration strategy is used. When using a prior integration, scaling analyte measurements suitably within each omic approach is especially important. In addition, the multi-omics datasets’ sample origin influences which integrative strategy can be employed. A prior integration necessitates the collection of data in the same biospecimens such as tissue, blood, etc., or individuals in order to match measurements to the same sample, whereas a post-integration does not. It is not possible to examine direct links between genes and metabolites and how they may relate to phenotype when the study is performed on the same individual but distinct biospecimens, such as genomic data from blood and metabolomic data from urine. Despite this limitation, it is possible to determine if data fit one biological paradigm; in this example; metabolites may serve as biomarkers for what is occurring at another level (i.e., genome) or alternatively, one omic modality can be used to orthogonally confirm biological pathways discovered by another omic modality [131].

Many human-focused studies have increasingly turned to the use of multiomics techniques (e.g., integration of genomic, transcriptomic, proteomic, and metabolomic platforms) to find relevant and accurate biomarkers. For example, in the past decade, multiomics studies of atrial fibrillation have identified a number of potential biomarkers of this condition [132]. Another study investigating the results of a metabolome and transcriptome-wide association study to identify genes influencing the human metabolome, found that this integration can support the causal role of ALMS1 (Alstrom syndrome 1) gene expression levels on N-acetylated compound concentration, whereas for HPS1 (Hermansky-Pudlak syndrome), a negative feedback loop between its expression levels and TMA (Trimethylamine) using an untargeted approach in nuclear magnetic resonance spectroscopy (NMR) and methylation quantitative trait loci (mQTLs) analysis. Multi-omics integrative analyses have also found use in investigateions of nutrition and functional food components [133], as well as in deciphering regulatory networks for complex disease traits [134]. Previous research has shown that a systems-level multi-omics investigation can provide more robust and valuable insights into biological mechanisms than a single platform analysis, especially where the condition under study can arise from a range of different underlying pathological mechanisms. Li, et al. [135] used advanced metabolomics and transcriptomics approaches to identify molecular and metabolic pathway abnormalities that could contribute to canine degenerative mitral valve disease (DMVD) development and progression. The goal of their study was to find pathways that could be altered with nutritional or pharmaceutical interventions to prevent, reverse, or control DMVD. While there are few studies of this depth in the veterinary field at present, we expect that integrative approaches showcasing multi-omics research in veterinary sciences will become increasingly important and prevalent over coming years, facilitating efficient biomarker discovery and development.

## 3. Challenges to Successful Biomarker Development

The example biomarker studies described in this review have been performed in different species and focus on diagnostics and monitoring of a wide range of conditions. While many are considered promising, the bulk of the biomarkers identified have yet to be developed into commercial products or diagnostic tests in practice. In many cases, the full suite of necessary validation steps required for a biomarker to enter clinical use has not been conducted or has not been published. Further, it appears that the rate of successful completion of biomarker studies to clinical use is higher for human medical applications than for the veterinary field. Although many of the same challenges persist in both human and veterinary medicine, some issues are more pronounced in the veterinary field and may help explain the deficits in this translational pipeline. Understanding and subsequently addressing these challenges is key to the future success of biomarker science in this field and realisation of the associated improvements in animal production efficiency and welfare they promise. 

### 3.1. Time and Finance towards the Biomarker Discovery

One of the foremost challenges is the time and expense required for establishing a biomarker. Research funding remains a major constraint in biomarker development, as extensive studies with a large sample size are required to ensure that any given biomarker is not only associated with, but truly able to predict, the clinical outcome [136]. Additionally, translating those identified markers into clinical practice requires further time and investment. Importantly, continuity of funding is essential as the path from discovery to clinical application is likely to take many years and interruptions in this process can compromise the ultimate success of even the most promising biomarkers. One example is the use of urinary estrogens to diagnose pregnancy in giant pandas, where researchers have been analysing estrogen metabolites as markers of pregnancy and viable cub development [137]; studies working towards this biomarker began a decade ago, characterising the biological and technical aspects and demonstrating the value of estrogen as a biomarker [137,138,139]. However, estrogen metabolites are yet to be validated and to undergo the clinical trials needed to confirm their utility as a robust test in zoo medicine practice and such studies are expected to take several additional years. 

Furthermore, there are unique challenges to the development of biomarkers for veterinary medicine. A given biomarker may need to be qualified multiple times, once for each applicable species. Other limitations relate to sample handling requirements, and difficulties in establishing cut-off values owing to breed differences. Historically, many animal biomarkers have relied on previous experiences from human medicine to reduce the time taken for many studies, exploiting already established methodologies and making modifications according to species-specific requirements. This application is very limited but useful if implemented with care and sufficient knowledge of species variation of the particular biomarkers. Examples of such scenarios include N-terminal pro b-type natriuretic peptide (NT-proBNP) and cardiac troponin T (cTnT): cardiac biomarkers used in human medicine that have been adapted to evaluate systemic inflammatory response syndrome (SIRS) in dogs. These biomarkers are well established markers in diagnosing cardiac dysfunction and evaluating prognosis in human medicine. Since cardiac dysfunction secondary to systemic inflammation has been reported in human medicine known as myocardial hibernation, increased concentration of these markers reported in SIRS in humans has been associated with myocardial hibernation [16,17]. Cardiac hibernation has been reported in dogs with experimentally induced sepsis, and more recent data suggest that plasma concentrations of cardiac biomarkers are increased in dogs with SIRS [140,141]. Thus, it has been hypothesised that these cardiac biomarkers may help in the diagnosis of cardiac dysfunction and the evaluation of prognosis in people with SIRS, and may also be useful to evaluate dogs with SIRS [142]. Indeed, NT-pro BNP and cTnT were found to be significantly increased in dogs with SIRS regardless of underlying diseases. Additionally, this study confirmed that the cTnT concentration was associated with survival in dogs with SIRS. Studies investigating the correlation of cardiac biomarkers with echocardiographic findings and inflammatory cytokines in canine patients with SIRS are warranted. Evidently, the background of research performed for human applications provides a huge advantage for the development of this biomarker in animal applications and its translation to clinical veterinary practice, reducing both time and funding required. 

A similar approach is to directly use commercially available kits of identified biomarkers to validate their use in veterinary medicine. Cardiac troponin I (cTnI) is also a peripheral blood biomarker for myocardial diseases in humans and veterinary medicine [82,84,127]. There are commercially available kits to monitor the cTnI levels in humans. It has been suggested that commercial human cTnI assays could be used in horses, cattle, and sheep and some assays have been evaluated and validated for use in camelids [101], cattle [99,143], horses [144] and goats [145]. Another example is biomarker identification for treatment monitoring in leishmaniosis in dogs. This research identified apolipoprotein A1 (APO-A1) as a potential biomarker using 2-dimensional electrophoresis followed by mass spectrometry analysis, observing that concentration of APO-A1 was low in dogs with leishmaniosis and increased with good response to treatment. From a panel of 8 differentially expressed proteins in leishmaniosis, selection of the APO-A1 biomarker was mainly based on its ability to be easily measured by an established automated immunoturbidimetric method and on previous work indicating its value as a possible diagnostic/prognostic biomarker of visceral leishmaniasis in humans [146]. Whilst incorporating expertise from other species, in particular from human medicine, can be advantageous in terms of time and finance, care must be taken to ensure that such biomarkers are truly the most appropriate and useful option for clinical decision making. Furthermore, in many situations human markers cannot be directly applied to veterinary medicine, not only because of differences in physiology, but also pragmatic aspects such as ease of collection of sample type (e.g., in many species collecting urine is more difficult than taking a blood sample), and clinical priorities (e.g., where patient comfort is prioritised over remission of neoplastic conditions or herd health is more critical than individual prognosis). Another example is the not-yet-elucidated maternal recognition of pregnancy (MRP) signal of horses, a factor that remains elusive despite being a fundamental component of reproductive biology. The review of Swegen [147] clearly shows how the biological and physiological differences between horses and other species pose challenges leading to lagging diagnostics in the early pregnancy of horses. The review also elaborates how the time and technological advances are accumulating knowledge towards pregnancy biomarker identification. Recent omics approaches including the proteomic analysis of early equine embryo secretome, blastocele fluid and capsule [148], uterine fluid of pregnant mares [149] together with transcriptomics studies [150,151] have provided more positive insight for the biomarker discovery to address the gap. Truly novel markers will require the full suite of discovery and validation studies in a given species and relevant clinical setting. Researchers, clinicians and funding bodies must work closely together to ensure clear goals, realistic expectations and thorough planning for all phases of biomarker development.

### 3.2. Requirement of Standardised Methodologies

Evidently, the scale and screening capacity of ‘omics platforms provide unparalleled appeal for biomarker discovery. However, sophisticated equipment and rapidly evolving technology mean that developing appropriate protocols for sample collection, processing and analysis is not always straightforward, nor consistent between studies. A thorough understanding of multiple components is required, e.g., biochemistry for sample preparation, analytical chemistry for instruments, and computational biology for data analysis. Thus, it is an essential requirement to have well-established standard methodology for each of these steps. Across all the omics platforms, collection and preparation of samples must be treated as a delicate process that necessitates close care to avoid sample handling bias [89]. In case–control studies any variation in sample selection and processing can result in systematic bias [20,152,153,154,155]. As an example, variation in sampling times between different studies can yield different results and it is important to take this into account when interpreting omics results or planning further studies. 

As a more established platform, proteomics has engaged in some interdisciplinary collaboration to standardise methods [156,157,158], while the lipidomics community is now initiating international standardisation [158]. The following steps have been suggested to minimise systematic bias [156].

*1*. *Sample selection*
  - Avoiding the pooling of samples
  - Not using a combination of blood plasma and serum, but instead consolidating around the exclusive use of a single substrate
*2*. *Collection of Samples*
  - Blood collection and pre analytical procedures should be standardised
  - Blood collection-to be conducted by the same person,
  - Pre-analytical procedures–use of identical conditions for centrifuging, containers, storage temperatures and times
  - Immediately centrifuging blood to generate plasma
  - Immediately harvesting plasma after centrifugation
  - Not withdrawing the last 500 μL of plasma at every possible stage to avoid contamination with platelets. If not, incorporating a second centrifugation clean-up step
  - Immediately freezing samples after harvesting

Furthermore, apart from these guidelines there are some standard analytical and statistical methods in use with a level of understanding in the international proteomics community [71,142,143]. Though some of these can apply to other omics approaches such as sampling standards, each field will need to agree on and publish clear standards in line with the relevant platforms’ requirements [158].

Likewise, metabolomics platforms are yet to develop universal standards for methods in biomarker discovery. As in other fields, those operating at the highly specialised end of the spectrum may be not fully aware of the clinical setting and requirements, while clinicians are unfamiliar with the technical aspects of metabolomic tests as well as the biochemical interdependencies of metabolites. Effective collaboration across the full spectrum of translation is clearly essential for successful development of truly useful assays. With increasingly complex datasets and a clear role for pattern recognition and machine learning, new statistical methodologies must be investigated for some study designs [110]. Towards this goal, there is ongoing improvement of the livestock metabolome database (LMBDB, available at http://www.lmdb.ca accessed on 27 July 2022), however further refinement of this dataset and its annotation promises to facilitate future untargeted metabolomics studies by increasing the number of identified metabolites. Nevertheless, for NMR-based approaches there remains considerable variability in the reference methods used for calibration. In brief, metabolite identification relies heavily on the mass spectrometry expertise of the operator and is further constrained by the availability of standards and as such, many of the inventories of promising markers contain only putative identifications. Thus, standardising the methods used within and across countries and laboratories remains a major challenge [159].

In addition to technical aspects and sample preparation, the standardisation and detailed reporting of experimental circumstances and animal traits are essential, and individual species should have their own guidelines [160]. Due to the snapshot nature of the results provided from most omics approaches, there remains a general problem of extrapolating findings to other scenarios, species, etc. Although it is nearly impossible to standardise experimental conditions and replicates across the entire range of veterinary species spanning from large to small animals, important information such as age, lactation number and stage, body condition, diet composition and feeding regimen, as well as a detailed description of sampling procedures and timing relative to physiological state, can be reported. Computer based algorithms can then be harnessed to model/integrate data from papers reporting on the proteome and other omics of animal diseases and facilitate the assembly of an atlas of all proteins and other biological agents present, regardless of their abundance or statistical analysis. Most proteomics and lipidomics investigations do not provide absolute quantitative data; they instead report patterns, such as an increase or reduction in comparison to internal standards. Although such studies have contributed substantially to our understanding of the pathophysiology of diseases, using proteomic techniques to study metabolic diseases has not yielded a comprehensive and consistent list of biomarkers. In the context of metabolic diseases, it could be argued that metabolomics has greater potential to identify suitable biomarkers. Thus, producing a cohesive atlas that includes all previously detected biological markers across independent studies, along with methods and animal data, will be very useful for accurate identification of markers in future studies. 

The multivariate outcomes from omics techniques, in accordance with computational and data requirements, necessitate significant bioinformatics resources, which are generally available online [113]. The identification of a limited number of potential candidates from thousands of proteins quantified by untargeted MS proteomics for downstream verification and validation using targeted assays is one of the rate-limiting phases in protein biomarker development [160]. Despite the fact that MS-based discovery platforms may measure a huge number of proteins they are frequently performed with a small number of samples, resulting in the “big p, small n” dilemma [161]. Thus, protein markers identified from discovery data may not be generalisable to independent datasets due to the limited sample size of typical discovery research. This issue arises frequently in omics-based association research, and it is frequently addressed using dimension reduction techniques such as principal component (PC) analysis and its supervised equivalents [162]. Because each PC is a linear combination of all original properties, predictive models based on PCs require genome-wide measurements as inputs and hence cannot be used as tailored clinical diagnostics but can be used to identify proteins serving as the main contributors to the predictive model and flag these for further investigation. As we can see from the summary details (Table 1) several novel cluster selection approaches and statistical methodologies are being developed to assist in these processes. Algorithms developed by Shi, Wen, Gao and Zhang [160] allowed for functional interpretation of the detected markers and should facilitate a smooth transition to the verification and validation platforms. Additional bioinformatics tools to assist modelling and validation are expected to emerge in the near future and will contribute significantly to the efficiency of the biomarker discovery process.

### 3.3. Determining the Clinical Utility of Biomarkers

Following the discovery of a biomarker and development of an accurate, statistically robust test, substantial hurdles remain in terms of establishing clinical relevance. These hurdles include evaluation of biological variability, diagnostic accuracy and diagnostic performance of the biomarker. All biochemical analytes display inherent biological variability, a crucial factor to consider in interpreting the diagnosis and developing cut-off values or guidelines. Indeed, both intraindividual (within-subject) and interindividual (between-subject) variation can impact biomarker performance in practice [163].

The estimated sensitivity (Se) and specificity (Sp) of a test are commonly used to evaluate its diagnostic accuracy [164], and the importance of each will vary according to the condition in question and clinical purpose of the assay. In some cases, there is a need for very high sensitivity while specificity may be lower; for example, in screening tests where a condition can be ruled out using the biomarker. On the other hand, in detecting conditions where diagnosis may result in high-risk procedures or culling, high specificity is required and therefore a very low rate of false positives is acceptable [165,166,167]. Optimising one of the Se or Sp by changing the cut-off value will result in decreasing the other as there is an inverse relationship between these two. A cut-off-independent method is provided by Receiver Operating Characteristic (ROC) analysis, which thereby prevents the loss of information compared to the traditional method of reporting Se and Sp at a single arbitrary cut-off value. ROC analysis summarises the diagnostic accuracy of a test across all possible operating points as pairs of 1-Sp and Se. For estimation of these pairs a gold standard is required, yet unfortunately such standards simply do not yet exist, or are not practicable, for many animal diseases owing to limitations in the clinical setting or pragmatic and financial constraints. Unless further information on the accuracy of one or more of the tests can be provided, the problem is not statistically identifiable in the absence of a gold standard. Consequently, methods for estimating ROC curves in the absence of a gold standard are required. For this reason, many researchers have used the Bayesian technique, i.e., using a likelihood method to link the data from the current evaluation study with prior information such as expert opinion and/or previously published data to provide posterior estimates of the estimated ROC curve and the Area Under the Curve [168]. Further, positive and negative predictive values (PPV and NPV) are determined for evaluating the diagnostic performance of a biomarker [169]. If the prevalence of the disease or condition in the population being tested is known, the predictive values of a diagnostic test can be calculated. When utilising a biomarker to identify a patient’s illness status, predictive values are essential factors in clinical practice because they can help select the patient population in which a specific test is likely to give meaningful results. Because PPV and NPV are dependent on disease prevalence, studies reporting these values should be critically evaluated and interpreted with caution if predictive values are estimated based on study prevalence, as this may not reflect disease prevalence in the population for which the test will be used. The disease prevalence in the study may differ from the disease prevalence in a specific clinical setting, and only a few diseases’ true prevalence is known in veterinary medicine [165,170] as the lack of gold standard tests in veterinary medicine affects this method as well. 

Using multiple markers (i.e., a ‘panel’) of the same type or a combination of types, i.e., proteins, DNA, RNA, lipids, and metabolites, will likely offer more information than the traditional measurement of a single marker. As clinical detection methods become simpler and more accessible, panel-type assays will be a welcome development, considering that most systemic conditions are indeed a complex matrix of biochemical parameters rather than a dramatic change in a single measurement. Examining patterns rather than single markers is expected to generate more powerful diagnostic assays and could provide additional information, such as disease staging or prognosis [129,130]. Thus, studies should aim at the identification of a panel of candidate biomarkers, which may collectively improve the sensitivity and specificity for detecting the disease. One such study [170] has shown that incorporating several inflammatory biomarkers associated with disease process alongside functional biomarkers describing digestive, absorptive, and secretory capacity of the gastrointestinal tract and degree of dysbiosis into an algorithm may provide an improved strategy to manage dogs with chronic inflammatory enteropathies in the future. 

A major point for consideration in the design of veterinary biomarkers is the cost–benefit ratio. Distinct from medical practice, the health of animals is often attributed economic value and the costs of treatment are generally covered directly by animal owners rather than health insurance or public health systems. In some cases, the health of the herd or public health outcomes must be prioritised over individual health. This will, in turn, impact the type of diagnostic assay that is considered affordable in any given clinical situation. In some instances, empirical treatment or culling are an economically preferable option to definitive diagnosis/treatment. In cases where herd health or reproductive performance are prioritised, the situation is reversed as relatively expensive but rapid and definitive diagnosis of a single animal may protect the health (or prevent culling) of an entire population or limit economic losses for a whole breeding season. Thus, it is crucial to consider the clinical context and decision-making that will surround the use of a given biomarker early in the discovery process, particularly aiming to ensure that the type of biomarker being sought is compatible with translational detection methods appropriate to the clinical setting.

### 3.4. Selecting a Point of Care Test to Use in Clinical Setup

While omics platforms provide remarkable discovery power, the ultimate destination for many biomarkers is incorporation into simple and rapid test protocols that can be performed in-house at a clinic, farm or field setting. Notably this is not an essential requirement for all biomarkers; some will be useful as send-away tests to be conducted in central laboratory facilities, especially for genetic screening and other non-time-critical scenarios. However, since the goal of many biomarkers is to improve speed and simplicity of diagnosis, selecting an appropriate method for how the biomarker can be detected at point of care (POC) is a crucial part of the development process. Point of care tests need to be simple to perform and cost-efficient, and to satisfy the sensitivity and specificity requirements appropriate for the clinical condition [171]. If chosen well, POC tests are a simple, quick, and generally inexpensive way to shorten hospital stays, reduce complications, and improve medication adherence. Test ordering, sample transfer to laboratories, and data reporting can all be reduced by using POC tests. Several high-throughput automated technologies have made it possible to introduce a wide range of tests that may be conducted quickly and easily, i.e., without the need for highly trained staff and without the need for laboratory processing [172]. 

Small bench-top analysers and hand-held devices are the two basic types of POC testing formats accessible in the clinical setting. Small bench-top analysers are essentially a downsized version of mainframe central lab equipment, with a few key differences to avoid operator error and offer quick, repeatable results. Handheld devices are created by employing cutting-edge microfabrication techniques that essentially combine many crucial analytical procedures, such as sample preparation, separation, analysis, and data reporting. Immunoassay methods are commonly used in devices for cardiac biomarker POC testing [172]. The most common formats for these devices are 2-site immunometric techniques, lateral-flow technology, and flow-through immunoassay systems. Rapid advancements in the field of antibody-based biosensors, on the other hand, are expected to usher in a new era in POC test development [173]. To offer the best quality diagnostic services, efficient management of POC test networks necessitates standardisation and integration of dedicated resources, policies, and multidisciplinary commitment and cooperation [171]. Emerging POC diagnostic instruments benefit from precision miniaturisation, perform as well as central laboratory methods in terms of analytical performance [174], and can accommodate multiplexed POC test biomarker panels. In reality, because of the significantly reduced number of sample-handling stages, POC testing has a real chance of improving analytical precision.

New and emerging technologies are enhancing the range of POC possibilities and the range of analytes that can be readily detected but selecting the appropriate and most compatible test is not always straightforward and may require significant investment into trials and optimisation. This must be considered when planning research and development particularly as a biomarker approaches clinical use/commercialisation phases. In addition, many of the newer technologies, e.g., microfabrication and microfluidics-based applications and those that combine multiple steps, have been developed commercially and are subject to intellectual property protection. This emphasises the need for researchers and clinicians to integrate with industry and pursue extensive collaboration in order to see biomarker projects realise their maximum potential.

### 3.5. Challenges Specific to ‘Omics Platforms

#### 3.5.1. Transcriptomics

Despite recent breakthroughs in RNA biomarker research, circulating RNAs have struggled to make it into the clinic, owing to their inconsistent specificity and repeatability concerns as biomarkers under various physiological and pathological situations. As previously mentioned, one of the key concerns is that RNA is generally unstable at ambient temperature due to the possibility of destruction by ribonucleases (RNase). As a result, both endogenous and exogenous RNases can impact the detection accuracy by degrading the target RNA over time throughout the incubation phases. The use of an RNase inhibitor in the experiment, as proven by Frei et al. [175], is one possible solution to this problem. However, RNAs found in numerous vesicles and biological components, such as exosomal RNAs, are protected by the exosome’s membrane structure and thus rendered inaccessible to RNases [176,177].

The choice of sample source and preparation process have a significant impact on RNA detection efficiency [178]. Wang et al. [179] demonstrated that the expression level of miRNA can differ between a person’s serum and plasma. Clinically relevant RNA concentrations in tissues, serum, and other bodily fluids are extremely low. As a result, a highly sensitive and specific approach for collecting RNAs from bodily fluids must be developed. Because a small disagreement in the analysis could result in false-positive diagnoses, the RNA extraction procedure is also critical. This was also demonstrated in work by McDonald et al. [180], who found that the extraction technique was responsible for the majority of the variance in RNA detection. Furthermore, physicochemical analysis and sequencing has revealed that RNAs from the same family frequently share identical properties [181]. As a result, the background response from closely comparable sequences of nontarget RNAs can readily compromise the accuracy and sensitivity of RNA detection. Natural variation in RNA expression levels across and within subjects is a significant challenge in RNA detection in clinical samples, which can be caused by changes in gender, race, age, diet, and other factors. It has also been discovered that when the sample size is small (less than 100 people), the variation is greater; consequently, the variation can be reduced by recruiting a large sample cohort [77]. For reliable detection, selecting the appropriate extraction method, as well as careful optimisation (i.e., incubation duration, centrifugation speed, etc.) of the extraction steps, is required [54].

#### 3.5.2. Proteomics 

Proteome-based biomarker discovery benefits from a well-established analytical platform, extensive characterisation and bioinformatics resources, and a suite of widely available methods of validation based on the relative stability of proteins and their antigenic properties. Traditionally, MS-based findings are validated using antibody-based techniques. This means that verification of novel biomarkers has relied heavily on the availability or production of suitable antibodies [113,182] for use in immunoassays such as immunoblotting, ELISA, immunohistochemistry, and flow cytometry. Antibody reagents with adequate specificity and sensitivity to assess new protein biomarkers are not always readily available, particularly where the protein is of interest is species-specific or novel/poorly characterised. Technical difficulties in multiplexing immunoassays for panels of biomarkers, as well as the high cost and extensive development time required to produce high-quality immunoassay reagents present further obstacles [113]. 

The specificity of antibodies is a limiting factor in many antibody-based approaches, and species-specific changes in amino acid sequence may limit the application of antibodies generated against the target protein in other species [113]. Furthermore, the performance of antibodies in various experimental setups can vary. Immunoassays such as ELISA have good quantitative power, however building a reliable ELISA system is time-consuming. Setting up a system with a working antibody is frequently thought to be faster in immunoblotting, which has recently become a de facto prerequisite for publishing as an orthogonal validation method for quantitative proteomics data [183], but when working quantitatively, complete validation is also required, and finding changes as small as 2 to 4-fold may be impossible [184]. Because of these constraints, “biological validation” is frequently performed to assess the matching mRNA concentration, preferably from the same sample, for validating proteomics results. However, because the quantity of mRNA does not always correspond to the abundance of the encoded protein [185], relying on a direct, absolute correlation between protein and mRNA levels is not a failsafe validation assessment [186]. Nonetheless, despite its flaws, assessing mRNA abundance is still the only method available in many circumstances [185], and it is limited to cells and tissues where both mRNA and proteins are accessible. Only protein, not mRNA, is available when proteomics is performed in bodily fluids such as saliva, urine, and blood serum. Optimistically, expanding commercial interests in manufacturing antibodies and assays specific for many animal species, including cattle, may soon fill this gap [113].

#### 3.5.3. Metabolomics

Being a more recently developed approach, metabolomics has yet to gain the popularity of proteomics and transcriptomics in the biomarker discovery field. Because of the intimate link between individual metabolites, a metabolomic biomarker differs from a protein biomarker and transcriptome biomarkers. Although there may be patterns of abundance that represent a disease state, the components assessed in other “omics” technologies are independent. Meanwhile a metabolome reveals co-related metabolites that change at the same time; the interdependence of metabolites results in a disease signature. As a disease progresses, the “signature” may change. A random change in a single metabolite will not produce a misleading signal because a metabolomic biomarker is a meta-biomarker. As a result, metabolomic signatures can be an effective tool for tracking changes in an individual’s health over time [110]. The major limitation presently is that significant artificial intelligence/algorithm work is associated with this form of signature/pattern detection, and this may not be easy to translate to a point-of-care test. On the other hand, species-specificity is less of a challenge in metabolomics than proteomics, as metabolic pathways are largely conserved across species [109]. The putative identification of metabolites, which very often cannot be confirmed, and small groups of samples, decrease the credibility of the metabolomics tools, as does the choice of controls and the presence of confounding factors; all of which can have a significant impact on assay outcomes. Such limitations explain, at least in part, why studies conducted for the same disease, with the same type of sample, and using the same analytical instruments frequently yield different results [187].

Another current issue in metabolomics is that there are two broad analytical approaches (NMR versus MS coupled with LC/GC) and the choice of platform appears to strongly affect the number and type of metabolites detected. NMR generates highly reproducible information on the identity and quantity of a limited number of metabolites, while MS is more sensitive and less reproducible [188]. Accordingly, in most cases MS-based biomarkers cannot be translated directly to clinical applications and additional work is needed to build robust and sensitive kits that can be used easily at the point of care. Metabolite validation, demonstration of clinical value, and development of suitable clinical procedures to make their measurement simple and reliable will therefore all be required before metabolite biomarkers may be used in clinical settings. 

The potential for metabolites as diagnostic markers to reach the clinic and make a significant difference in patient health is enormous provided these obstacles can be addressed, which will hopefully take place over the next several years as the analytical and bioinformatic platforms continue to evolve and become more widely accessible. Once these research-to-clinic barriers have been overcome, metabolome-based biomarkers are expected to significantly improve diagnoses, staging, prognosis, and therapy for a variety of diseases [91,92,95,173,175,176].

#### 3.5.4. Lipidomics

The primary challenges in lipidomics-based biomarker discovery relate to the intrinsic complexity of lipids as a biochemical class and to the relative immaturity of the network of tools used to interpret lipidomics outputs. Lipids are an enormously diverse biochemical category, encompassing hundreds of thousands of different molecules with widely ranging structural and physicochemical properties. Ultimately, this means that even so-called ‘untargeted’ lipidomics techniques cannot include all the classes of lipids in a single analysis, and that there is no true shotgun approach; instead, different extraction, processing and analytical methods must be chosen based on the lipid classes of interest, or multiple analyses conducted. This evidently has consequences for the cost and logistics of conducting screening studies for lipid biomarker discovery. The other implication of this diversity is that characterisation of lipids detected by MS can be a complex undertaking. Lipidomics does not benefit from the same direct relationship that exists between proteins and their coding genes as unique identifiers; instead, lipidomic signatures emerge as patterns in mass and retention times, while the precise molecular identity of each lipid species is often ambiguous. Structural validation and absolute quantification are then required using targeted techniques and internal standards. Isotopic species and the formation of adducts can further complicate this process [189]. While pioneering groups in the field have put forward recommendations to ensure quality and consistency of data generation [189,190], the field is yet to reach consensus and, further, analytical technology platforms and lipid structure databases are constantly evolving requiring updated guidelines generated at a relevant pace. Beyond biomarker discovery, deriving optimal value from lipidomics data also means effectively integrating the lipidomics output with associated biological pathways and metabolic networks. Due to discrepancies in identifiers and in quality of annotation bet22ween data and metabolic networks, mapping experimentally measured lipid molecules onto metabolic networks is currently difficult. Researchers are addressing this by making their methods publicly and freely available in an open-source format. Ideally, both the metabolomics/lipidomics and network modelling communities must annotate metabolites with both ontology identifiers (e.g., ChEBI) and chemical representations (InChIKeys, SMILES) [191,192].

Beyond the discovery stage, a key consideration in the lipidomics field is how a putative biomarker can be detected in a clinical setting. Whereas proteins can be quite consistently detected using immunoassay-based approaches, the detection of lipids is less straightforward, and the specific biochemical properties of each lipid class will determine the options available for detection at POC. While clinical MS is an option, this would often entail major investment, specialised staff/training, and involved sample preparation. This can only be justified in scenarios where large numbers of samples are to be analysed and is unlikely to be a suitable point-of-care approach in the veterinary field. Portable and benchtop NMR-based sensors are under development for clinical application but are not yet at a stage where resolution and sensitivity are adequate for reliable quantitative detection of individual lipid species [193]. Alternative options include the design of enzymatic assays or biosensor technology exploiting the optical, electrochemical or mechanical properties of the lipid species in question [194]. This, in turn, requires appropriate expertise in lipid biochemistry and engineering, as well as significant time and funding for the design, prototyping and optimisation of such devices. This phase of biomarker development may be difficult to plan for since the final lipid classes of interest revealed by screening studies cannot be known at the outset, making resource requirements impossible to predict. Expanding knowledge of the normal and pathological lipidomes across different tissues and species and their integration with other ‘omics datasets in the future will alleviate this challenge to some extent. Future biomarker screening studies should balance retaining their discovery aspect with guidance by existing data and can be hypothesis-driven; prior knowledge about a biological process will help predict which lipid classes are most likely to be quantitatively altered and therefore to hold promise as biomarkers. 

## 4. Conclusions and Future Directions

Biomarker discovery is clearly a rapidly expanding research area in the veterinary field. Successful biomarker discovery, development and translation is being facilitated by emerging technologies but remains challenging due to several factors such as financial support, species variations, a smaller number of samples in some species, difficulty in sample collection, and lack of method standardisation or bioinformatics resources with which to analyse the output of some platforms. Identifying such challenges early in the research and development planning process can help to overcome many obstacles and maximise the chance of success of such projects. Here, we also emphasise the critical need for close collaboration between clinicians, researchers and funding bodies and the need to set clear goals for biomarker requirements and realistic application in the clinical setting, ensuring that biomarker type, method of detection and clinical utility are compatible, and adequate funding, time and sample size are available for all phases of development. We contend that embracing these approaches will assist in many of the promising preliminary biomarkers attaining their full potential in clinical application, ultimately reducing workload in veterinary practice, enhancing animal welfare outcomes, and improving economic viability of livestock industries.

## Figures and Tables

**Table 1:** Summary of ‘omics platforms and their role in biomarker discovery (Refer to the Supplementary Table S1 for detailed description of bioinformatical and statistical methods, sample numbers and validation methods).

Omics Platform	Reference	Species	Concern	Result	Main Technology
Genomics	Meurs et al., 2007	Dog-Doberman Pinscher	Familial Dialated Cardiomayopathy (DCM)	Demonstrated that DCM in the Doberman Pinscher dogs is a familial disease inherited as an autosomal dominant trait.	Polymerase Chain Reaction (PCR), Sequencing
	Brooks et al., 2010	Horse-Arabian foal	Lavender Foal Syndrome (LFS)	Identified a frameshift mutation in the MYO5A gene that leads to Lavender Foal Syndrome in the Egyptian Arabian breed of horse.	PCR, Sequencing, and Genotyping
	Neibergs et al., 2014	Holstein calves	Bovine respiratory disease complex (BRDC)	Identified common genomic regions associated with BRDC susceptibility that can be further characterized and used for genomic selection.	Genomic Wide Association Analysis-SNP identification-qPCR
	Arendt et al., 2015	Dogs-Golden retrievers	Genetic associations between Canine Mast Cell Tumours (CMCT).	Identified a SNP associated with development of CMCT in the GNAI2 gene and a candidate mutation that resulting in a truncated protein.	Genotyping of SNP- PCR, Illumina 170K canine HD SNP arrays
	Menzi et al., 2015	Holstein cattle	Cholesterol deficiency	A mutation represents a 1.3kb insertion of a transposable LTR element (ERV2-1) in the coding sequence of the APOB gene,	Genotyping, Sanger sequencing
Transcriptomics	Barrey et al., 2010	Horses	To identify miRNA candidates in the muscles of control and affected horses suffering from polysaccharide storage myopathy (PSSM) and recurrent exertional rhabdomyolysis (RER).	A specific miRNA profile was related to each myopathy: a higher expression of *mir-1, 133, 23a, 30b, 195 and 339 in RER-TF* vs. control-TF a higher expression of *mir-195* in PSSM-Cob vs. control-Cob.	Real-Time Polymerase Chain Reaction (RT-PCR)
	Desjardin et al., 2014	Horses	Equine cartilage and subchondral bone miRNAs and suggest their involvement in osteochondrosis (OC) physiopathology	Observed miRNAs differentially expressed between healthy and OC cartilage and bone.	Next-generation sequencing
	Dirksen et al., 2016	Dogs	Distinguish between parenchymal, biliary, and neoplastic hepatobiliary ds	Demonstrated a micro-RNA panel consisting of *miR-21*, *miR-122*, *miR-126*, *miR-200c*, and *miR-222;* distinguishing between the parenchymal, biliary and neoplastic hepatobiliary ds	Reverse Transcription and RT-QPCR
	da Costa Santos et al., 2018	Horse Warmblood cross	Equine Insulin Resistance	Results demonstrated different miRNA profiles between two groups: Insulin sensitive (IS) and Insulin resistant (IR)	Microarray, RT-QPCR
	Lecchi et al., 2019	Water buffaloes	Brucella infection	*miR*-let-7f, *miR-151*, *miR-30e*, *miR-191*, *miR-150* and *miR-339b*	Next Generation Sequencing
Proteomics	Kuleš et al., 2014	Dogs	Identification of dogs naturally infected with *Babesia canis canis*	Confirmed two dominant pathogenic mechanisms of babesiosis, haemolysis and acute phase response which may be helpful in future biomarker studies.	Two-dimensional electrophoresis (2DE), Electrospray Ionisation Mass Spectrometry
	Mudaliar et al., 2016	Cow	Bovine milk in an experimental model of Streptococcus uberis mastitis:	2552 non-redundant bovine peptides were identified, and from these, 570 bovine proteins were quantified	On-line reversed-phase liquid chromatography and mass spectrometry (LC-MS),
	Martinez-Subiela et al., 2017	Dogs	Identification of biomarkers for treatment monitoring in canine leishmaniosis	Identification of new serum proteins that significantly change in concentration after canine leishmaniosis treatment.	Tandem Mass Tag (TMT), LC-MS
	Escribano et al., 2019	Sheep	Identification of possible new salivary biomarkers of stress in sheep- identify biological pathways and proteins differentially expressed in the saliva proteome.	4 new metabolic pathways and 13 proteins differentially represented in the saliva of sheep after an application of acute stress.	TMT incorporated LC−MS/MS
	Ploypetch et al., 2019	Dogs	Canine oral tumours	SENP7, TLR4 and NF-κB as potential salivary biomarkers of canine oral tumours.	MALDI-TOF MS
	Liu et al., 2020	Cats	Congestive heart failure (CHF) due to primary cardiomyopathy	27 proteins differentially regulated in feline CHF.	Tandem Mass Tag (TMT), LC-MS
	Lazensky et al., 2021	Florida manatee (*Trichechus manatus latirostris*)	Investigating an increase in Florida manatee mortalities	Identified proteins that were differentially expressed in the serum of manatees affected by two distinct mortality episodes.	2D-DIGE and isobaric tags for relative and absolute quantification (iTRAQ) LC–MS/MS.
Metabolomics	Hailemariam et al., 2014	Cow	Identifying postpartum or periparturient disease states in dairy cows.	Found (carnitine (C0), propionyl carnitine (C3), and lysophosphatidylcholine acyl C14:0 (lysoPC a C14:0), 4 wk before parturition and phosphatidylcholine acyl-alkyl C42:4 and phosphatidylcholine diacyl C42:6 could be used to discriminate healthy controls from diseased cows 1 wk before parturition	Targeted quantitative metabolomics approach
	Wu et al., 2020	Poultry	Mycoplasma gallisepticum (MG) and *Escherichia coli* (E. coli) co-infection model in respiratory disease in poultry	Co-infection induces distinct alterations in the serum metabolome owing to the activation of Arachidonic Acid (AA) metabolism. LTC4 in serum could be used as the biomarker for detecting poultry respiratory disease.	Non-targeted metabolomics LC-MS system
Lipidomics	Christmann et al., 2019	Horses	Evaluation of asthma caused by exposing to hay	cPA 16:0 and DAG 36:2 were 2 novel lipid mediators identified in surfactant obtained from asthmatic horses with clinical disease.	Shotgun lipidomics on ion-trap mass spectrometer.
	Rivera-Velez et al., 2019	Cats	Determine the effects of repeated meloxicam administration on the feline plasma and urine lipidome.	Identified lipids in plasma urine that could serve as biomarker candidates.	Untargeted approach-liquid chromatography– quadrupole time-of-flight mass spectrometry approach. (LC-QTOF-MS)
	Koelmel et al., 2019	Fish	Investigation of wildlife mass mortality events- affected by pancreatitis	1000-fold increase in ceramides and correlated with disease severity	UHPLC system in positive and negative ion mode.
	Ceciliani et al., 2021	Cow	Subclinical mastitis	Influence of NAS-IMI [Inflammatory Infection (IMI)caused by non-aureus staphylococci (NAS)] on the milk lipidome.	Untargeted approach- LC-QTOF-MS
	Jackeline et al., 2021	Dog	Lipid Biomarkers for diagnosis and disease progression of canine atopic dermatitis (CAD)	A feature selection strategy found oleic acid containing triacylglycerides, long-chain acylcarnitines and sphingolipids as predictive lipids that highly correlated (R2 = 0.89) with the disease severity score of patients.	MRM- LC-QTOF-MS equipped with a Jet Stream ESI ion source -rapid lipid-profiling mass spectrometry
Multiomics	Li et al., 2015	Dog	Identify nutritional targets for Degenerative Mitral Valve Disease (DMVD)	Data suggested that the fatal gene program hypothesis, wherein the stressed heart switches to anaerobic metabolism, decreasing fatty acid oxidation, and increasing glycolysis may apply also to dogs.	Metabolomics- LC-MS Transcriptomics- RNA-seq, RT-qPCR

## Supplementary Materials

The following supporting information can be downloaded at: https://www.mdpi.com/article/10.3390/ani12172194/s1, Table S1: Summary of some Omics examples in veterinary medicine.
